# Prognostic Values for the mRNA Expression of the ADAMTS Family of Genes in Gastric Cancer

**DOI:** 10.1155/2020/9431560

**Published:** 2020-08-20

**Authors:** Liang Liang, Jin-hui Zhu, Gang Chen, Xin-gan Qin, Jun-qiang Chen

**Affiliations:** ^1^Department of Gastrointestinal Surgery, The First Affiliated Hospital of Guangxi Medical University, Nanning, Guangxi Zhuang Autonomous Region 530021, China; ^2^Department of Pathology, The First Affiliated Hospital of Guangxi Medical University, Nanning, Guangxi Zhuang Autonomous Region 530021, China

## Abstract

The “A Disintegrin and Metalloproteinase with Thrombospondin Motif” (ADAMTS) family of genes is involved in the occurrence and development of different cancers. However, the prognostic value of these genes in gastric cancer (GC) has not been revealed. The present study was thus conducted to determine the prognostic value for the ADAMTS family of genes in GC. First, we evaluated the mRNA expression levels of the ADAMTS family in GC patients using a GEPIA dataset. Thereafter, we determined the prognostic value of these genes by analyzing their mRNA level using the Kaplan–Meier Plotter database. The mRNA expression level of ADAMTS12 was randomly validated by qRT-PCR and meta-analysis while its coexpression genes were derived using Coexpedia. Finally, we performed Gene Ontology (GO) annotation and Kyoto Encyclopedia of Genes and Genomes (KEGG) pathway enrichment analyses using the OmicShare Tools. Compared to normal tissues, expression of *ADAMTS2* and *12* was significantly higher while that of *ADAMTS1*, *13*, and *15* was significantly lower in GC tissues. According to the RNA-seq and gene chip data, the ADAMTS family (*6*, *7*, *12*, *15*, and *18*) of genes was closely related to the prognosis of GC, and their high expression levels were associated with poor prognosis and survival time. In addition, *ADAMTS12* was highly expressed in 20 pairs of GC tissues based on RT-PCR (*P*=0.016) and meta-analysis (SMD: 0.73, 95% CI: 0.32–1.14, *P* < 0.001). GO and KEGG pathway analyses indicated that the *ADAMTS12* coexpressed genes were enriched in the pathways of extracellular matrix organization, extracellular matrix structural constituent, extracellular matrix, and protein digestion and absorption. Herein, we discovered the prognostic values and biological roles of the ADAMTS genes in GC.

## 1. Introduction

Gastric cancer (GC) is the most common lethal cancer worldwide. Although it is ranked fifth based on the number of patients diagnosed each year, it has the third highest mortality rate of all cancers [1]. Although the techniques used for early screening have improved, and there have been considerable improvements in comprehensive prevention and treatment measures owing to surgical treatment [2, 3], the prognosis of GC remains unsatisfactory [2]. Therefore, establishing reliable biomarkers to predict the prognosis of GC could contribute to the development of individualized clinical treatment.

Carcinogenesis is associated with abnormalities in different cellular and intercellular mechanisms such as extracellular matrix (ECM) remodeling. Among all ECM proteases, “A Disintegrin and Metalloproteinase with Thrombospondins” (ADAMTSs) are a relatively new group and are considered to be associated with carcinogenesis and the local/distant spread of cancer [4].

ADAMTS is a complex extracellular protease related to carcinogenesis and tumor protection. ADAMTS can cleave or interact with different ECM components or regulatory factors, ultimately affecting cell adhesion, migration, proliferation, and angiogenesis. Differences in the overexpression, mutation, or epigenetic silencing of the ADAMTS genes were identified in different sources of tumors, indicating the direct effect of these metalloproteinases on the development of cancer [5].

Presently, only few studies have been carried out on the ADAMTS family in GC. Jiang found that *ADAMTS2* may be a potential biomarker for determining the prognosis of GC. This researcher also demonstrated that *ADAMTS1*, *8*, and *18* were highly expressed in GC and its lymph node metastasis [6]. Conversely, however, other studies have found that the hypermethylation of *ADAMTS8* and *18* is related to a decrease in the expression of GC and may play an important role in the invasion and metastasis of this malignancy [7]. *ADAMTS12* may also play a role in the tumor process owing to its proteolytic activity or serve as a potential molecule involved in the regulation of cell adhesion [8].

Because the role of the ADAMTS family of genes in tumor diseases remains unclear, especially in the study of prognosis, we aimed to determine the prognostic value of their expression in GC.

## 2. Materials and Methods

### 2.1. Detection of the Gene Expression of the ADAMTS Family

The expression level of the ADAMTS family of genes was detected using the GEPIA tool (http://gepia.cancer-pku.cn) [9]. Normal samples were selected from TCGA and GTEx databases and compared to those from the TCGA tumor group, using a standard processing pipeline. To explore the expression of the ADAMTS family of genes in the different stages of tumor development, major pathological stages or subpathological stages were selected for plotting. All expression data were first log_2_(TPM + 1) transformed for plotting. One-way ANOVA was employed for differential analysis, and disease state (tumor or normal) was used as a variable to calculate the differential expression. The RNA-seq data for stomach cancer, which were used in the heatmap, were downloaded from UCSC Xena project (http://xena.ucsc.edu). Genomic alterations of the ADAMTS family were analyzed by cBioPortal, an integrative analytic platform of TCGA [10].

### 2.2. Prognostic Analysis of the ADAMTS Gene Family in GC Using the Kaplan–Meier (K-M) Plotter

The K-M plotter database was established using gene expression data and survival information from Gene Expression Omnibus (GEO) and TCGA (http://kmplot.com/analysis) [11]. Briefly, the gene chip data from the GEO database and mRNA-seq data from the TCGA database were selected. The chip data contains GSE14210, GSE22377, GSE51105, GSE15459, GSE29272, GSE62254, and other chips. The clinical parameters of tumor subtypes (tumor stage and grade) were recorded for patients, including gender, treatment, and HER2 status. The prognosis of ADAMTS in these subtypes was then compared. Thereafter, 19 genes were separately searched in the K-M plotter and the resulting images were downloaded from the website.

### 2.3. Verification of *ADAMTS12* Expression Using the Chip Data and RT-PCR

We searched the chip data to identify the GC-related chip datasets, including cancer and its control group, and extracted the expression of *ADAMTS12*. The STATA v12.0 statistical software was employed in the meta-analysis to derive the continuous variables. Standardized mean difference (SMD) and 95% confidence interval (CI) were calculated using the Random (I–V heterogeneity) test. A two-sided *P* value <0.05 was considered to indicate statistical significance.

Following the retrieval of written informed consent, a total of 20 paired GC and corresponding adjacent noncancerous tissues were collected from patients who underwent gastric resection at the Department of Gastrointestinal Surgery between January 2019 and May 2019 at the First Affiliated Hospital of Guangxi Medical University. All experiments were approved by the Ethics Committee of the Hospital. Total RNA extraction was conducted with NucleoZOL (Macherey-Nagel, Germany), following the protocol of the manufacturer. Reverse transcription of the extracted RNA and real-time quantitative PCR (qRT-PCR) were successively performed according to the instructions provided in the RT-PCR kit (TaKaRa Biotechnology) and the fluorescence quantitative PCR kit (TaKaRa Biotechnology). The primer sequences for ADAMTS12 and *β*-actin were produced by Sangon Biotech Company (Shanghai, China). The following primer sequences were used: ADAMTS12, 5′-AACGCTATCGCTTGTGCAAC-3′ and 5′-CTCACAAGGATGTGCTGGGT-3′; and *β*-actin, 5′-TGGCACCCAGCACAATGAA-3′ and 5′-CTAAGTCATAGTCCGCCTAGAAGCA-3′. The relative expression levels of ADAMTS12 were quantified using the 2^−ΔΔCT^ method.

### 2.4. Gene Function Enrichment Analysis

Coexpedia is a database that contains context-associated coexpression networks inferred from individual series of microarray samples for human and mouse from chip data. Hence, this database (http://www.coexpedia.org) was employed to analyze the *ADAMTS12*-related genes [12]. In addition, we analyzed the *ADAMTS12* coexpressed genes using RNA-seq data to determine the possible mechanisms of action of ADAMTS12. Kyoto Encyclopedia of Genes and Genomes (KEGG) and Gene Ontology (GO) analyses were performed using the OmicShare tools, a free online platform for data analysis (http://www.omicshare.com/tools).

## 3. Results

### 3.1. mRNA Expression Level of the ADAMTS Family in GC

**Figure 1:**
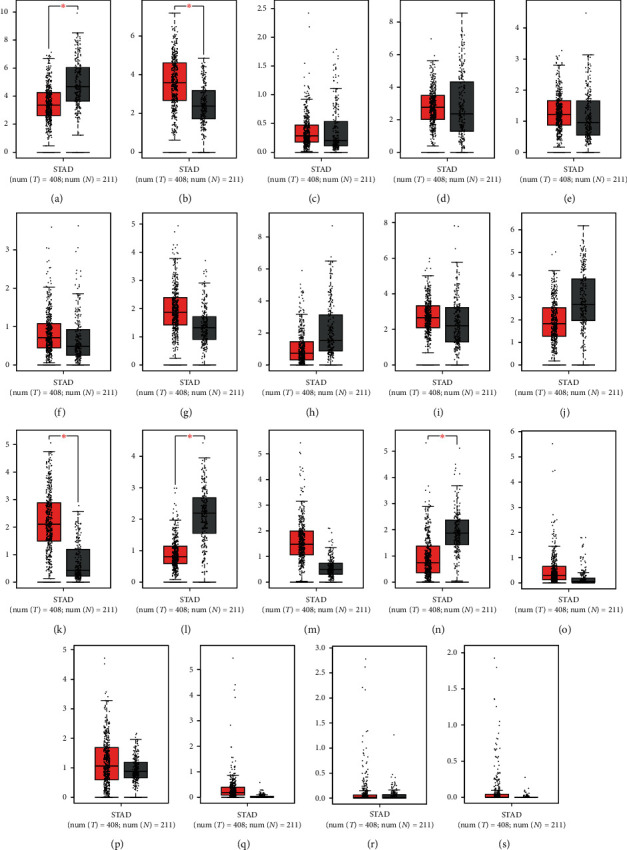
The expression of the ADAMTS family based on sequencing data. The mRNA expression of the ADAMTS family between tumor and normal; red: tumor; gray: normal; ^*∗*^*P* < 0.01. (a) ADAMTS1, (b) ADAMTS2, (c) ADAMTS3, (d) ADAMTS4, (e) ADAMTS5, (f) ADAMTS6, (g) ADAMTS7, (h) ADAMTS8, (i) ADAMTS9, (j) ADAMTS10, (k) ADAMTS12, (l) ADAMTS13, (m) ADAMTS14, (n) ADAMTS15, (o) ADAMTS16, (p) ADAMTS17, (q) ADAMTS18, (r) ADAMTS19, and (s) ADAMTS20.

**Figure 2:**
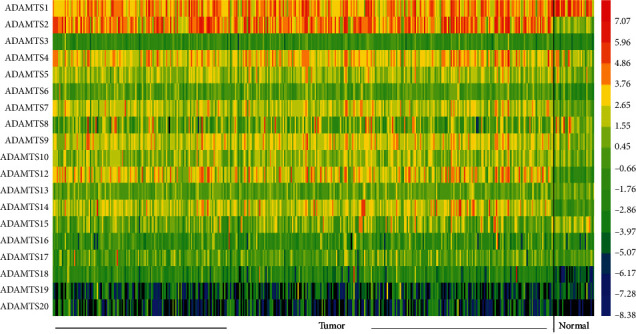
Heatmap depicting the expression of the ADAMTS family in GC patients. Red represents high expression; blue represents low expression; black represents not detected.

**Figure 3:**
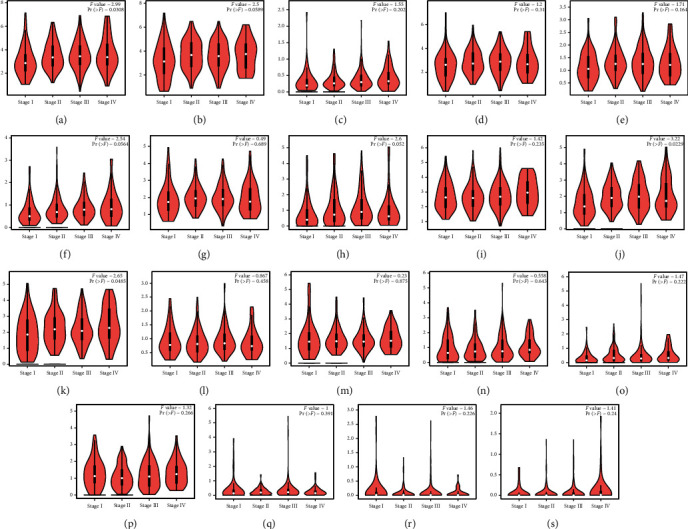
Expression of the ADAMTS family at different clinical stages. One-way ANOVA was performed to calculate the expression of the ADAMTS family in the differential pathological stage. (a) ADAMTS1, (b) ADAMTS2, (c) ADAMTS3, (d) ADAMTS4, (e) ADAMTS5, (f) ADAMTS6, (g) ADAMTS7, (h) ADAMTS8, (i) ADAMTS9, (j) ADAMTS10, (k) ADAMTS12, (l) ADAMTS13, (m) ADAMTS14, (n) ADAMTS15, (o) ADAMTS16, (p) ADAMTS17, (q) ADAMTS18, (r) ADAMTS19, and (s) ADAMTS20.

**Figure 4:**
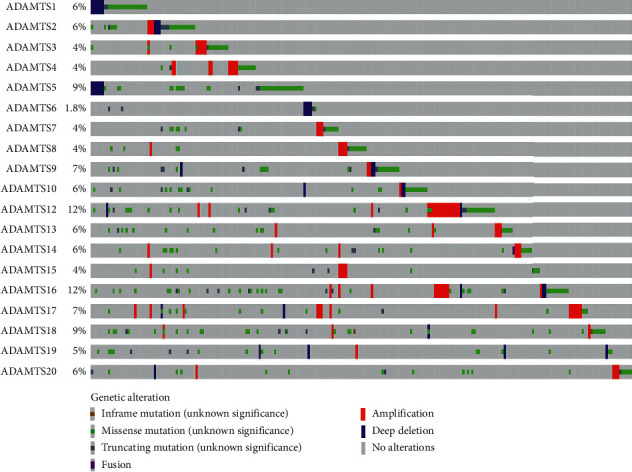
The genomic alterations of the ADAMTS family in RNA-seq data. *ADAMTS12* was found to have the highest alteration (12%), with amplification detected to be the main cause of alterations.

Based on our search of the RNA-seq data, the expression level of *ADAMTS2*, *ADAMTS9*, *ADAMTS12*, *ADAMTS14*, and other genes in the GC group was higher than that found in the normal group (Figure 1) (*P* < 0.05). However, the expression level of *ADAMTS1* was significantly lower in the GC tissues than in normal tissues (*P* < 0.05) (Figure 1). The heatmap presented in Figure 2 shows the expression of each ADAMTS family of molecules in tumor tissues and the adjacent tissues.  Figure 3 depicts the relationship between the ADAMTS molecules and tumor progression. Genes such as *ADAMTS1*, *ADAMTS10*, and *ADAMTS12* were found to increase with tumor progression (*P* < 0.05). By analyzing the ADAMTS family of genes, we found that *ADAMTS12* and *ADAMTS16* had the highest mutation rate (i.e., 12%), which was mainly due to amplification and missense mutation. The lowest rate of genetic change occurred in *ADAMTS6* (rate = 1.8%; Figure 4).

### 3.2. Prognostic Values of the ADAMTS Family in Patients with GC Based on RNA-Seq and Chip Data

**Figure 5:**
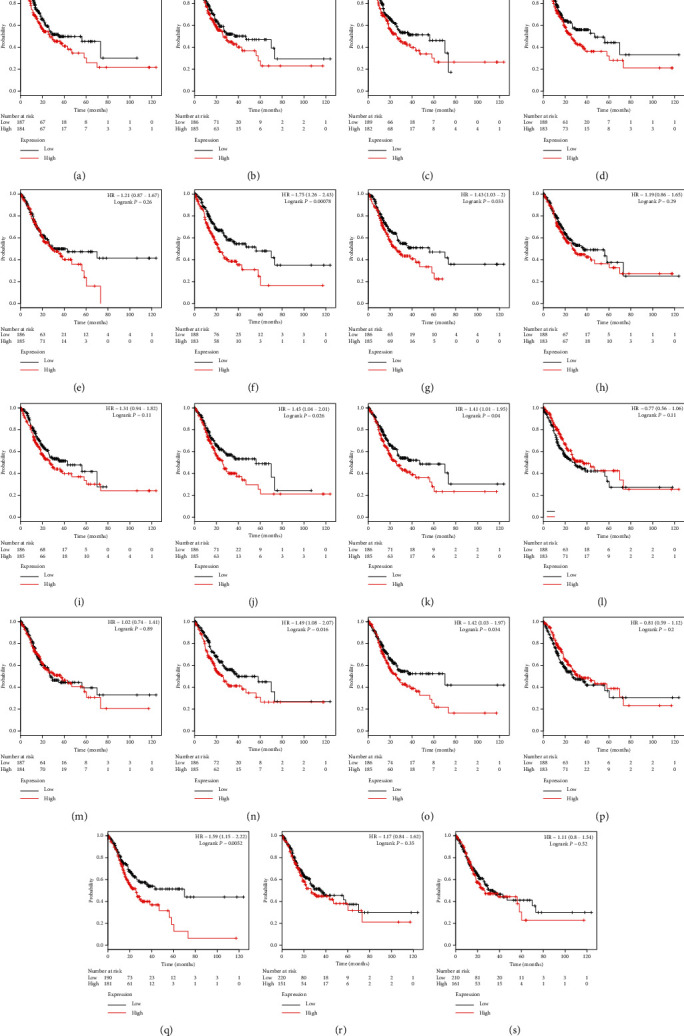
The overall survival value for the expression of the ADAMTS family based on RNA-seq data. (a) ADAMTS1, (b) ADAMTS2, (c) ADAMTS3, (d) ADAMTS4, (e) ADAMTS5, (f) ADAMTS6, (g) ADAMTS7, (h) ADAMTS8, (i) ADAMTS9, (j) ADAMTS10, (k) ADAMTS12, (l) ADAMTS13, (m) ADAMTS14, (n) ADAMTS15, (o) ADAMTS16, (p) ADAMTS17, (q) ADAMTS18, (r) ADAMTS19, and (s) ADAMTS20.

Using the RNA-seq data, we determined the prognostic value for the mRNA expression of the ADAMTS family in GC. *ADAMTS4* (HR = 1.39, *P* < 0.001), *ADAMTS6* (HR = 1.75, *P* < 0.001), *ADAMTS7* (HR = 1.43, *P* < 0.033), *ADAMTS10* (HR = 1.45, *P* < 0.026), *ADAMTS12* (HR = 1.41, *P* < 0.040), *ADAMTS15* (HR = 1.49, *P* < 0.016), *ADAMTS16* (HR = 1.42, *P* < 0.034), *ADAMTS18* (HR = 1.59, *P*=0.005), and other molecules were found to be associated with the overall survival (OS) of patients with GC. In fact, the survival time of patients with a high expression of these molecules was significantly shorter than that of patients with a low expression of these molecules (Figure 5).

**Figure 6:**
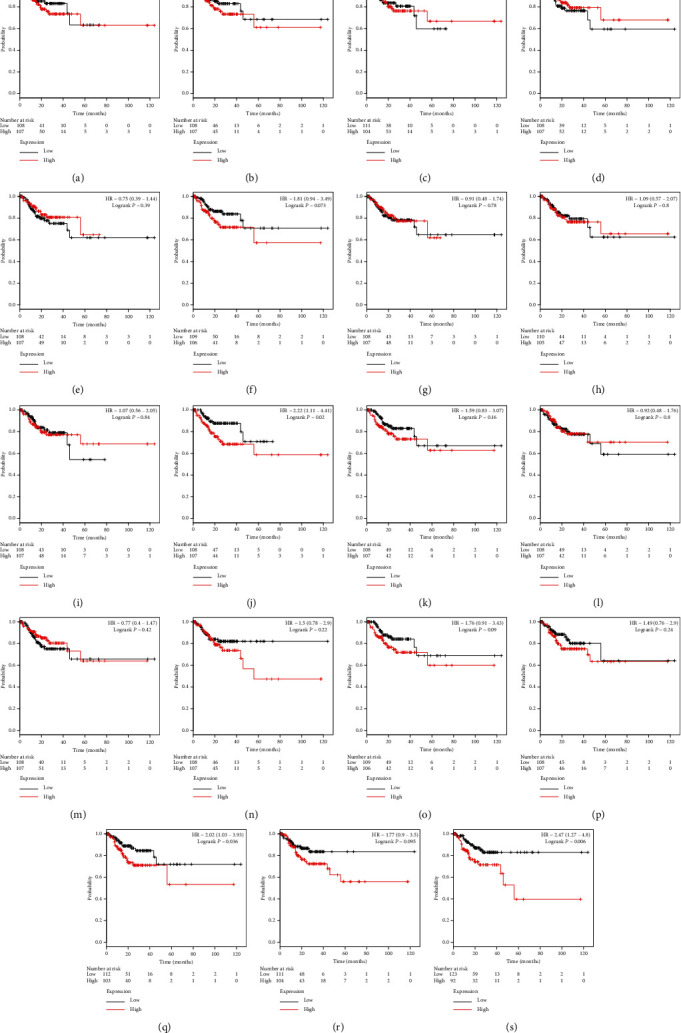
The relapse-free survival value for the expression of the ADAMTS family based on RNA-seq data. (a) ADAMTS1, (b) ADAMTS2, (c) ADAMTS3, (d) ADAMTS4, (e) ADAMTS5, (f) ADAMTS6, (g) ADAMTS7, (h) ADAMTS8, (i) ADAMTS9, (j) ADAMTS10, (k) ADAMTS12, (l) ADAMTS13, (m) ADAMTS14, (n) ADAMTS15, (o) ADAMTS16, (p) ADAMTS17, (q) ADAMTS18, (r) ADAMTS19, and (s) ADAMTS20.

The expression levels of *ADAMTS10* (HR = 2.22, *P*=0.020), *ADAMTS18* (HR = 2.02, *P*=0.036), and *ADAMTS20* (HR = 2.47, *P*=0.006) were recognized to be associated with the recurrence of GC. The survival time of patients with a high expression level of these genes was significantly lower than that of patients with a low expression level of these genes (Figure 6).

**Figure 7:**
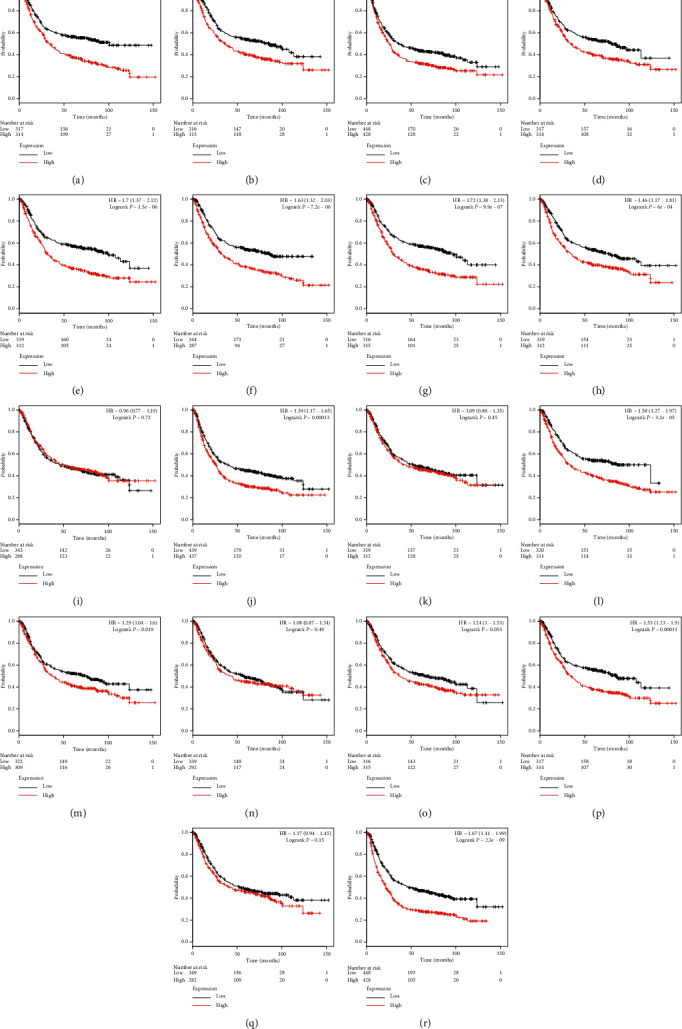
The overall survival value for the expression of the ADAMTS family based on chip data. (a) 222486_s_at ADAMTS1, (b) 226311_at ADAMTS2, (c) 214913_at ADAMTS3, (d) 235368_at ADAMTS5, (e) 237411_at ADAMTS6, (f) 228911_at ADAMTS7, (g) 235649_at ADAMTS8, (h) 1554697_at ADAMTS9, (i) 232133_at ADAMTS10, (j) 221421_s_at ADAMTS12, (k) 223844_at ADAMTS13, (l) 230167_at ADAMTS14, (m) 1553427_at ADAMTS15, (n) 238125_at ADAMTS16, (o) 1552726_at ADAMTS17, (p) 230040_at ADAMTS18, (q) 1553179_at ADAMTS19, and (r) 220717 ADAMTS20.

Finally, we verified the prognostic value of 19 genes of the ADAMTS family in the chip data. As a result, *ADAMTS1* (HR = 1.68, *P* < 0.001), *ADAMTS2* (HR = 1.49, *P* < 0.001), *ADAMTS3* (HR = 1.33, *P* < 0.001), *ADAMTS5* (HR = 1.4, *P* < 0.002), *ADAMTS6* (HR = 1.7, *P* < 0.001), *ADAMTS7* (HR = 1.63, *P* < 0.001), *ADAMTS8* (HR = 1.72, *P* < 0.001), *ADAMTS9* (HR = 1.46, *P* < 0.001), *ADAMTS12* (HR = 1.39, *P* < 0.001), *ADAMTS14* (HR = 1.58, *P* < 0.001), *ADAMTS15* (HR = 1.29, *P* < 0.001), *ADAMTS18* (HR = 1.53, *P* < 0.001), and *ADAMTS20* (HR = 1.67) were found to be closely related to OS in patients with GC (*P* < 0.001). The total survival time of patients with a high expression level of these molecules was significantly shorter than that of patients with low expression (Figure 7).

**Figure 8:**
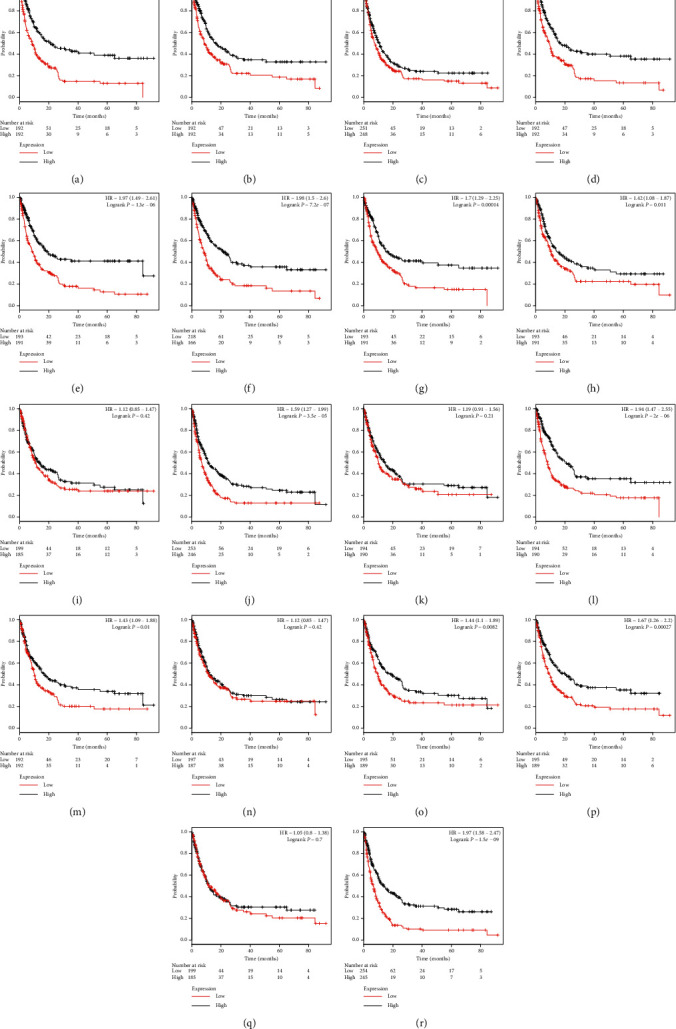
The postprogression survival value for the expression of the ADAMTS family based on chip data. (a) 222486_s_at ADAMTS1, (b) 226311_at ADAMTS2, (c) 214913_at ADAMTS3, (d) 235368_at ADAMTS5, (e) 237411_at ADAMTS6, (f) 228911_at ADAMTS7, (g) 235649_at ADAMTS8, (h) 1554697_at ADAMTS9, (i) 232133_at ADAMTS10, (j) 221421_s_at ADAMTS12, (k) 223844_at ADAMTS13, (l) 230167_at ADAMTS14, (m) 1553427_at ADAMTS15, (n) 238125_at ADAMTS16, (o) 1552726_at ADAMTS17, (p) 230040_at ADAMTS18, (q) 1553179_at ADAMTS19, and (r) 220717 ADAMTS20.

For postprogression survival (PPS) (Figure 8), we found that 14 genes, namely, *ADAMTS1* (HR = 2.14, *P* < 0.001), *ADAMTS2* (HR = 1.66, *P* < 0.001), *ADAMTS3* (HR = 1.27, *P* < 0.001), *ADAMTS5* (HR = 1.89, *P* < 0.001), *ADAMTS6* (HR = 1.97, *P* < 0.001), *ADAMTS7* (HR = 1.98, *P* < 0.001), *ADAMTS8* (HR = 1.7, *P* < 0.001), *ADAMTS9* (HR = 1.42, *P* < 0.001), *ADAMTS12* (HR = 1.59, *P* < 0.001), *ADAMTS14* (HR = 1.94, *P* < 0.001), *ADAMTS15* (HR = 1.43, *P* < 0.001), *ADAMTS17* (HR = 1.44, *P* < 0.001), *ADAMTS18* (HR = 1.67, *P* < 0.001), and *ADAMTS20* (HR = 1.97, *P* < 0.001), were closely related to PPS. The total survival time of patients with a high expression level of these genes was significantly shorter than that of patients with a low expression level.

Through subgroup analysis, genes such as *ADAMTS15*, *16*, and *18* were identified to be associated with unfavorable OS in stage II; *ADAMTS2*, *5*, *6*, *7*, and *20* were associated with unfavorable OS in stage III; and *ADAMTS1*, *5*, *6*, *7*, and *14* were associated with unfavorable OS in stage IV (Table 1). The expression of *ADAMTS1*, *5*, *6*, *7*, *12*, *16*, *18*, and *20* was associated with poor OS and did not significantly differ based on gender. Although the expression levels of *ADAMTS2*, *4*, *14*, and 15 were related to poor OS among men, only the expression level of *ADAMTS9* was associated with poor OS among women (Table 2).  Table 3 contains the prognosis of ADAMTS in the tumor stratification group. The expression of genes such as *ADAMTS6*, *7*, *12*, *15*, *16*, and *18* was closely related to poor OS in grade III.

### 3.3. Validation of ADAMTS12 mRNA Expression Level

**Figure 9:**
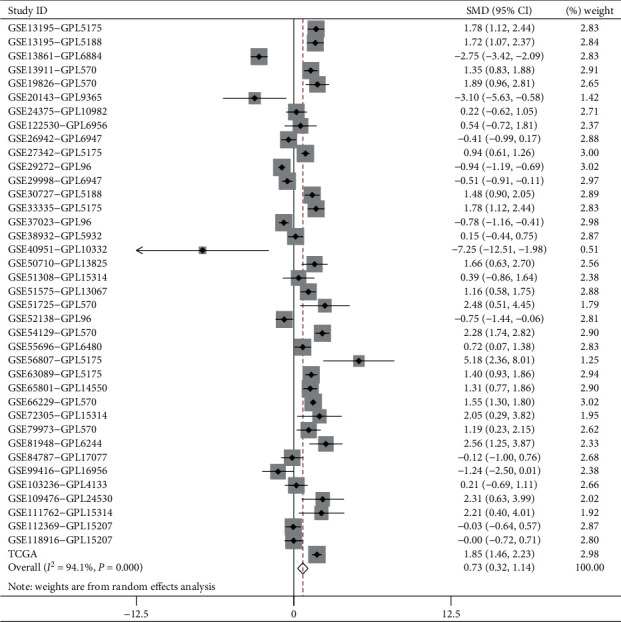
Forest plot of *ADAMTS12* expression level. Meta-analysis revealed that the expression level of *ADAMTS12* in the cancer patients was higher than that in noncancer patients (standardized mean difference (SMD): 0.73 (0.32–1.14), *P* < 0.001).

**Figure 10:**
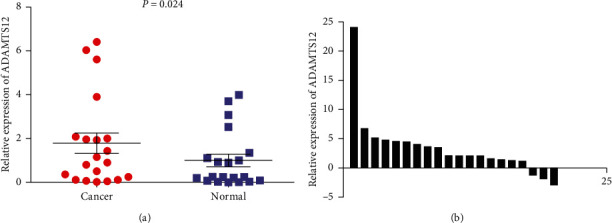
The expression of *ADAMTS12* in gastric cancer patients. The expression level of *ADAMTS12* was higher in GC tissues compared to that in adjacent noncancerous tissues (*P* < 0.05).

By screening 38 GC chips from the GEO database (a total of 1559 GC samples and 883 control samples), we found that the expression level of *ADAMTS12* was significantly increased in GC (SMD = 0.73, 95% CI (0.32–1.14) (*P* < 0.05) (Figure 9)). Thereafter, by performing qRT-PCR using 20 pairs of clinical samples, we found that the expression level of *ADAMTS12* in GC tissues was significantly higher than that in adjacent tissues (*P*=0.024) (Figure 10).

### 3.4. ADAMTS12 Functional Enrichment Analysis

**Figure 11:**
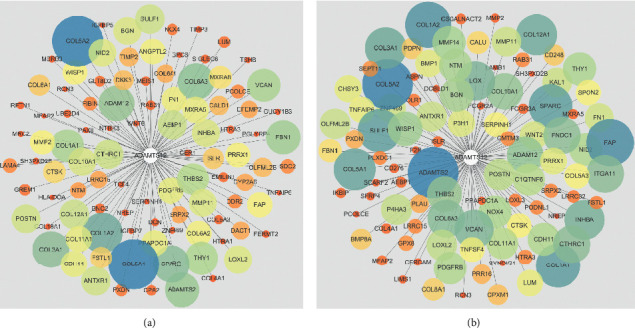
Coexpression networks. The coexpressed genes of *ADAMTS12* in (a) RNA-seq data and (b) chip data. In both network diagrams, color and size represent the degree of correlation, with the blue and large circles depicting a high degree of correlation.

**Figure 12:**
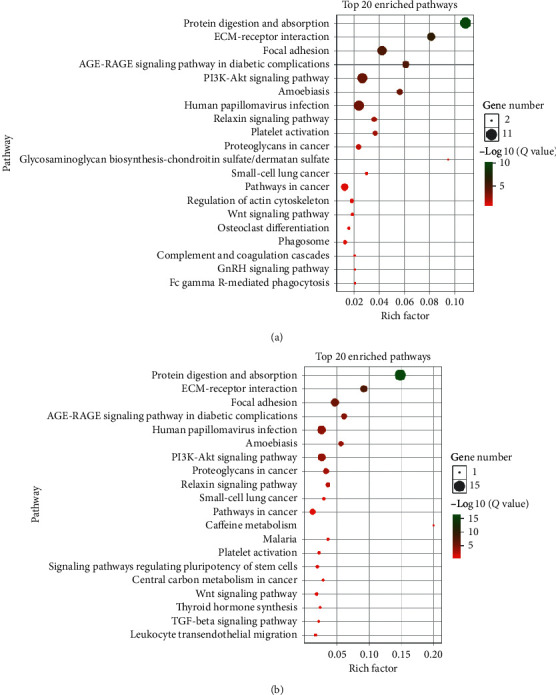
(a) KEGG analysis of the coexpressed genes from the RNA-seq data revealed that protein digestion and absorption was the most significantly enriched pathway. (b) KEGG analysis of the coexpressed genes from the chip data also revealed that the pathway of protein digestion and absorption was significantly enriched.

**Figure 13:**
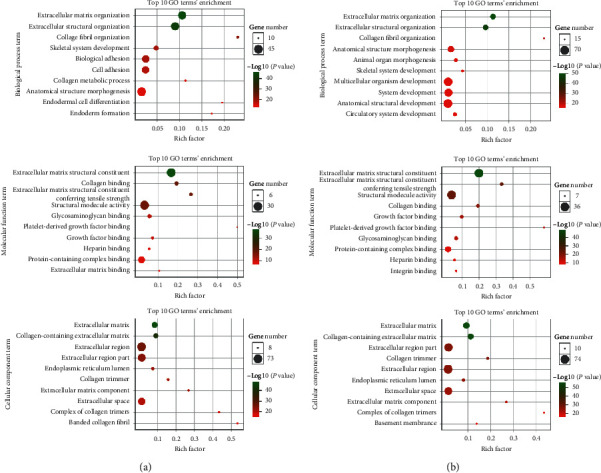
GO functional analysis of the coexpressed genes. The coexpressed genes of the (a) RNA-seq data and (b) chip data. The top 10 genes for biological process, molecular function, and cellular component term are displayed.

By analyzing the chip data in the Coexpedia database, we found that the A*DAMTS12* expression-related genes were *COL5A2*, *COL5A1*, *COL1A2*, *SPARC*, *COL3A1*, *COL6A3*, *ADAMTS12*, *ADAMTS2*, *FBN1*, *COL1A1*, and other related genes (Figure 11(a)). Similarly, by analyzing genes related to *ADAMTS12* expression using the RNA-seq data, we found that *ADAMTS2*, *COL5A2*, *FAP*, *COL1A2*, *COL1A1, SPARC*, *SULF1*, *COL5A1*, *COL3A1*, and *COL12A1* were the genes closely related to its expression (Figure 11(b)). Through functional enrichment analysis of the top 100 expression-related genes, we found that these genes were closely related to signaling pathways such as protein digestion and absorption, ECM-receptor interaction, and focal adhesion (Figure 12). Through GO analysis, we found that *ADAMTS12* and other related genes were significantly enriched in the ECM organization (biological process), ECM structural constituent (molecular function), and ECM (cellular component) (Figure 13).

## 4. Discussion

In the present study, we sought to explore the significance of the expression of the ADAMTS family of genes in the prognosis of GC by using a K-M plotter. Thus, the expression of this family of genes in GC was analyzed. According to the RNA-seq and gene chip data, most genes in the ADAMTS family (*6*, *7*, *12*, *15*, and *18*) were found to be closely related to the prognosis of GC. In addition, their high expression was found to be associated with poor prognosis and survival time. By exploring gene mutations of the ADAMTS family, we recognized that it was indeed the most frequent cause of gene alterations. Furthermore, through PCR analysis, we could confirm that the expression level of *ADAMTS12* in GC was consistent with the sequencing data.

Based on the studies conducted on the ADAMTS family, we recognized that most were related to osteoarthritis, blood vessels, and diseases of the platelet [13]. In the present study, however, we primarily discussed prior researches and the role of the ADAMTS family of genes in cancers.

*ADAMTS1* is highly expressed in pancreatic cancer and ovarian cancer [14, 15]. Although a study found that its expression level was elevated in GC [6], other studies have found that it displays a low expression level in this malignancy [16, 17]. As these findings align with the sequencing data obtained in our study, its expression level in GC is yet to be clarified. Through a study of *ADAMTS1* in breast cancer, its expression level was found to be low [18–21]. However, another study found that it can promote breast cancer metastasis [22].

*ADAMTS2* is highly expressed in GC and colon cancer and is closely related to distant metastasis and the prognosis of colon cancer. However, it is an independent prognostic factor for GC [23, 24]. We found that the overexpression of *ADAMTS2* was associated with unfavorable OS in GC patients, which aligns with the findings of the abovementioned study.

*ADAMTS3* is involved in the alteration of osteosarcoma matrices [25], and the single nucleotide polymorphism (SNP) of the *ADAMTS3* gene is an independent prognostic biomarker for cutaneous melanoma [26].

*ADAMTS4* is highly expressed in colon cancer and is associated with poor prognosis [27]. *ADMATS4* was also found to be mutated in early-onset familial colorectal cancer [28].

The role of *ADAMTS5* in digestive system tumors is unclear, but its expression level was confirmed to be low in GC, colon cancer, and liver cancer [29–31]. A study found that *ADAMTS5* overexpresses colon cancer cells to inhibit their invasion and migration [30]. However, Yu et al. found that it is highly expressed in colon cancer and is associated with lymph node metastasis [32]. A high expression level of *ADAMTS5* promotes the invasion and migration of human glioblastoma, non-small-cell lung cancer, and HNSCC cells [33, 34].

*ADAMTS6* is highly expressed in esophageal cancer and is an independent marker of its prognosis [35]. Notably, *ADAMTS6* displays the opposite trend in expression in colon and rectal cancer [36, 37]. In the current analysis, we found that a high expression level of *ADAMTS6* was significantly correlated with favorable survival in GC patients.

Presently, only few reports exist on the direction of *ADAMTS7* tumors. Among the genetic mutations in liver cancer, the Asian *ADAMTS7* mutation is only found in Asian Americans [38].

A study revealed that the *ADAMTS8* gene is highly methylated in GC while its mRNA expression level is significantly reduced [7]. *ADAMTS8* is underexpressed in liver cancer and affects its progression by targeting the ERK signaling pathway [39]. Conversely, it is highly expressed in head and neck squamous cell carcinoma [40]. Porter et al. demonstrated that the expression level of *ADAMTS8* is a predictor of poor OS [41].

*ADAMTS9* is poorly expressed in breast cancer, colorectal cancer, and GC and is associated with the hypermethylation of their promoters [42–44]. *ADAMTS9* can also inhibit tumor progression by inhibiting angiogenesis [45].

*ADAMTS12* is associated with ovarian and renal cancer metastases, and its expression level is significantly elevated in metastatic tumors compared to primary tumors [46, 47]. *ADAMTS12* is also highly expressed in esophageal squamous cell carcinoma [48] but has low expression in colon cancer [49]. A previous study revealed that *ADAMTS12* has anti-tumor-growth and angiogenesis effects [50]. In the present study, the data clearly indicate that a higher expression level of *ADAMTS12* was significantly correlated with poor prognosis in patients with GC.

Garam et al. found that *ADAMTS13* is a prognostic risk factor for colon and liver cancer [51]. The single nucleotide polymorphism of *ADAMTS14* is closely related to liver cancer and oral cancer [52, 53].

*ADAMTS15* inhibits tumor cell migration and angiogenesis in breast cancer and serves as an independent prognostic factor of this cancer type [41, 54]. *ADAMTS15* also has a negative correlation with the degree of tumor tissue differentiation in colorectal cancer and inhibits the growth and invasion ability of colon cancer cells [55]. According to our research, *ADAMTS15* has a low expression level in GC and may also exhibit an inhibitory effect.

The DNA of *ADAMTS16* displays a hypermethylation state in colorectal tissue and can inhibit tumor proliferation [56]. However, ADAMTS16 was found to be highly expressed in the esophagus, and its knockout inhibited tumor invasion [57].

*ADAMTS18* is a gene that has received considerable attention. In GC, colorectal cancer, pancreatic cancer, lung cancer, breast cancer, esophageal cancer, and nasopharyngeal carcinoma, its low expression level was found to be associated with the abnormal methylation of their promoter [58–63]. Similar studies have also found that *ADAMTS19* is highly methylated in colorectal cancer [64]. Evidently, *ADAMTS20* causes somatic mutation in GC patients with liver metastases [65]. In the present study, *ADAMTS18* was found to be correlated with unfavorable prognosis in GC patients.

In conclusion, the present study was carried out to determine the mRNA expression levels of the ADAMTS family of genes in GC and the prognostic value of their expression using a KM survival curve. As a result, the expression of the ADAMTS family of genes was found to be closely related to the poor prognosis of GC patients. Such findings could enable a better understanding of the prognostic function of the ADAMTS family of genes in GC. Furthermore, these findings may serve as a favorable predictor of GC prognosis, ultimately contributing to the design of strategies.

## Figures and Tables

**Table 1:** Correlation between genes expression of ADAMTS family and OS in gastric cancer patients of clinical stage.

ADAMTSs gene chip	Clinical stages I (n = 69); II (n = 145); III (n = 319); IV (n = 152)	HR (95% CI)	*P* value	ADAMTSs RNA-seq	Clinical stages I (n = 50); II (n = 111); III (n = 149); IV (n = 38)	HR (95% CI)	*P* value
ADAMTS1	I	1.95 (0.60–6.40)	0.26	ADAMTS1	I	1.08 (0.32–3.64)	0.90
	II	1.57 (0.83–2.97)	0.16		II	2.00 (0.98–4.05)	0.05
	III	1.11 (0.77–1.62)	0.57		III	1.12 (0.69–1.80)	0.65
	IV	1.52 (1.02–2.27)	0.04		IV	1.12 (0.49–2.56)	0.78

ADAMTS2	I	0.26 (0.07–0.96)	0.03	ADAMTS2	I	2.11 (0.61–7.25)	0.22
	II	0.62 (0.33–1.19)	0.15		II	1.62 (0.80–3.26)	0.17
	III	1.56 (1.07–2.28)	0.02		III	1.36 (0.84–2.19)	0.21
	IV	1.29 (0.87–1.91)	0.20		IV	1.28 (0.55–2.96)	0.57

ADAMTS3	I	1.18 (0.43–3.23)	0.75	ADAMTS3	I	0.60 (0.18–2.07)	0.42
	II	1.52 (0.83–2.76)	0.17		II	1.26 (0.63–2.51)	0.51
	III	1.13 (0.85–1.5)	0.40		III	1.35 (0.83–2.18)	0.22
	IV	1.46 (1.00–2.15)	0.05		IV	1.12 (0.48–2.58)	0.80

ADAMTS4(none)	—			ADAMTS4	I	1.42 (0.41–4.91)	0.57
					II	2.04 (0.99–4.19)	0.05
					III	1.24 (0.76–2.01)	0.39
					IV	1.20 (0.52–2.78)	0.67

ADAMTS5	I	0.48 (0.16–1.49)	0.19	ADAMTS5	I	0.69 (0.22–2.16)	0.52
	II	1.66 (0.86–3.18)	0.13		II	1.20 (0.61–2.36)	0.61
	III	1.81 (1.24–2.64)	<0.01		III	1.44 (0.89–2.33)	0.14
	IV	1.51 (1.02–2.25)	0.04		IV	1.32 (0.57–3.08)	0.51

ADAMTS6	I	0.41 (0.12–1.33)	0.12	ADAMTS6	I	1.15 (0.36–3.65)	0.81
	II	1.40 (0.74–2.63)	0.29		II	1.50 (0.75–3.00)	0.25
	III	1.36 (0.93–1.98)	0.11		III	1.68 (1.04–2.72)	0.03
	IV	1.31 (0.88–1.95)	0.18		IV	3.37 (1.30–8.69)	<0.01

ADAMTS7	I	2.56 (0.77–8.56)	0.11	ADAMTS7	I	2.82 (0.75–10.63)	0.11
	II	1.35 (0.71–2.57)	0.35		II	1.10 (0.55–2.17)	0.79
	III	1.77 (1.21–2.57)	<0.01		III	1.44 (0.89–2.33)	0.14
	IV	1.76 (1.18–2.63)	<0.01		IV	1.19 (0.51–2.76)	0.68

ADAMTS8	I	1.87 (0.56–6.23)	0.30	ADAMTS8	I	1.89 (0.57–6.32)	0.29
	II	1.52 (0.80–2.88)	0.19		II	1.16 (0.59–2.28)	0.66
	III	1.2 (0.82–1.74)	0.34		III	1.55 (0.96–2.52)	0.07
	IV	1.4 (0.94–2.09)	0.10		IV	1.13 (0.49–2.59)	0.77

ADAMTS9	I	0.86 (0.29–2.57)	0.78	ADAMTS9	I	1.11 (0.34–3.65)	0.87
	II	1.39 (0.74–2.61)	0.31		II	1.08 (0.55–2.13)	0.82
	III	1.36 (0.94–1.98)	0.10		III	1.22 (0.75–1.99)	0.41
	IV	1.58 (1.06–2.34)	0.02		IV	1.35 (0.58–3.12)	0.49

ADAMTS10	I	2.12 (0.69–6.48)	0.18	ADAMTS10	I	1.10 (0.33–3.62)	0.88
	II	0.89 (0.48–1.65)	0.71		II	1.72 (0.86–3.43)	0.12
	III	1.15 (0.79–1.67)	0.47		III	1.25 (0.77–2.02)	0.37
	IV	0.76 (0.51–1.13)	0.17		IV	1.46 (0.63–3.39)	0.38

ADAMTS12	I	1.06 (0.40–2.84)	0.91	ADAMTS12	I	1.41 (0.45–4.44)	0.56
	II	1.67 (0.92–3.05)	0.09		II	1.72 (0.85–3.46)	0.12
	III	1.33 (1.00–1.77)	0.05		III	1.43 (0.89–2.32)	0.14
	IV	1.13 (0.77–1.65)	0.54		IV	1.57 (0.67–3.66)	0.29

ADAMTS13	I	1.75 (0.57–5.37)	0.32	ADAMTS13	I	0.39 (0.12–1.31)	0.11
	II	1.33 (0.71–2.49)	0.37		II	0.99 (0.50–1.94)	0.97
	III	1.11 (0.77–1.62)	0.57		III	0.91 (0.56–1.46)	0.69
	IV	0.86 (0.58–1.28)	0.47		IV	0.88 (0.39–2.01)	0.76

ADAMTS14	I	0.83 (0.26–2.67)	0.75	ADAMTS14	I	0.66 (0.21–2.09)	0.47
	II	1.19 (0.62–2.25)	0.60		II	0.76 (0.38–1.49)	0.42
	III	1.42 (0.98–2.07)	0.06		III	1.36 (0.84–2.20)	0.21
	IV	2.10 (1.40–3.16)	<0.01		IV	0.96 (0.42–2.23)	0.93

ADAMTS15	I	1.13 (0.38–3.39)	0.82	ADAMTS15	I	0.69 (0.21–2.26)	0.54
	II	1.33 (0.71–2.49)	0.36		II	2.14 (1.05–4.35)	0.03
	III	1.10 (0.76–1.60)	0.62		III	1.32 (0.82–2.13)	0.26
	IV	1.31 (0.89–1.95)	0.17		IV	1.78 (0.76–4.17)	0.18

ADAMTS16	I	1.25 (0.42–3.73)	0.69	ADAMTS16	I	1.10 (0.35–3.50)	0.87
	II	1.00 (0.53–1.87)	0.99		II	2.60 (1.26–5.36)	<0.01
	III	1.06 (0.73–1.54)	0.76		III	1.13 (0.70–1.81)	0.62
	IV	1.20 (0.81–1.77)	0.37		IV	1.66 (0.71–3.88)	0.24

ADAMTS17	I	0.89 (0.29–2.70)	0.84	ADAMTS17	I	1.22 (0.38–3.87)	0.74
	II	1.88 (0.99–3.58)	0.05		II	0.53 (0.26–1.08)	0.08
	III	1.17 (0.81–1.70)	0.40		III	0.71 (0.44–1.14)	0.16
	IV	0.97 (0.65–1.43)	0.86		IV	1.58 (0.69–3.64)	0.27

ADAMTS18	I	1.32 (0.42–4.16)	0.64	ADAMTS18	I	2.51 (0.75–8.42)	0.12
	II	2.35 (1.20–4.59)	0.01		II	2.41 (1.18–4.82)	0.01
	III	1.06 (0.73–1.55)	0.74		III	1.49 (0.92–2.41)	0.11
	IV	1.22 (0.82–1.81)	0.32		IV	1.00 (0.43–2.33)	1.00

ADAMTS19	I	3.17 (0.97–10.37)	0.05	ADAMTS19	I	4.89 (1.32–18.14)	<0.01
	II	1.07 (0.57–2.01)	0.82		II	0.52 (0.26–1.06)	0.07
	III	1.06 (0.73–1.54)	0.74		III	1.30 (0.80–2.09)	0.29
	IV	0.97 (0.65–1.44)	0.88		IV	1.52 (0.65–3.52)	0.33

ADAMTS20	I	1.56 (0.55–4.37)	0.40	ADAMTS20	I	1.58 (0.48–5.21)	0.45
	II	1.36 (0.75–2.46)	0.31		II	1.44 (0.73–2.85)	0.29
	III	1.42 (1.07–1.89)	0.02		III	0.87 (0.54–1.41)	0.58
	IV	1.25 (0.85–1.82)	0.26		IV	1.04 (0.44–2.45)	0.92

**Table 2:** Correlation between genes expression of ADAMTS family and OS in gastric cancer patients of differentiation.

ADAMTSs gene chip	Differentiation poorly (n = 166); moderately (n = 67); well (n = 32)	HR (95% CI)	*P* value	ADAMTSs RNA-seq	Differentiation I (n = 12); II (n = 134); III (n = 218)	HR (95% CI)	*P* value
ADAMTS1	Poorly differentiated	0.85 (0.57–1.27)	0.43	ADAMTS1	Grade I	1.29 × 109 (0–inf)	0.23
	Moderately differentiated	0.96 (0.50–1.83)	0.90		Grade II	1.26 (0.72–2.20)	0.43
	Well differentiated	—			Grade III	1.37 (0.91–2.07)	0.13

ADAMTS2	Poorly differentiated	1.17 (0.78–1.74)	0.45	ADAMTS2	Grade I	1.29 × 109 (0-inf)	0.23
	Moderately differentiated	1.31 (0.69–2.52)	0.41		Grade II	1.35 (0.76–2.37)	0.30
	Well differentiated	—			Grade III	1.30 (0.86–1.97)	0.21

ADAMTS3	Poorly differentiated	1.23 (0.82–1.83)	0.32	ADAMTS3	Grade I	1.29 × 109 (0-inf)	0.23
	Moderately differentiated	1.35 (0.71–2.59)	0.36		Grade II	0.79 (0.45–1.40)	0.43
	Well differentiated	1.52 (0.64–3.61)	0.34		Grade III	1.33 (0.88–2.01)	0.17

ADAMTS5	Poorly differentiated	1.16 (0.72–1.88)	0.54	ADAMTS5	Grade I	1.29 × 109 (0-inf)	0.23
	Moderately differentiated	0.96 (0.50–1.84)	0.91		Grade II	0.98 (0.56–1.73)	0.95
	Well differentiated	—			Grade III	1.26 (0.83–1.91)	0.27

ADAMTS6	Poorly differentiated	1.07 (0.66–1.73)	0.78	ADAMTS6	Grade I	3.9 × 109 (0-inf)	0.44
	Moderately differentiated	1.49 (0.78–2.84)	0.23		Grade II	1.45 (0.81–2.58)	0.20
	Well differentiated	—			Grade III	1.93 (1.27–2.93)	<0.01

ADAMTS7	Poorly differentiated	0.97 (0.65–1.44)	0.86	ADAMTS7	Grade I	1.41 (0.08–23.57)	0.81
	Moderately differentiated	2.39 (1.24–4.59)	<0.01		Grade II	1.12 (0.63–1.99)	0.69
	Well differentiated	—			Grade III	1.58 (1.03–2.41)	0.04

ADAMTS8	Poorly differentiated	1.10 (0.68–1.79)	0.69	ADAMTS8	Grade I	3.56 × 109 (0-inf)	0.06
	Moderately differentiated	1.43 (0.75–2.74)	0.28		Grade II	1.00 (0.57–1.75)	0.99
	Well differentiated	—			Grade III	1.07 (0.71–1.61)	0.76

ADAMTS9	Poorly differentiated	1.07 (0.66–1.74)	0.77	ADAMTS9	Grade I	1.29 × 109 (0-inf)	0.23
	Moderately differentiated	1.34 (0.70–2.55)	0.38		Grade II	0.85 (0.48–1.49)	0.57
	Well differentiated	—			Grade III	1.50 (0.98–2.29)	0.06

ADAMTS10	Poorly differentiated	1.08 (0.67–1.74)	0.76	ADAMTS10	Grade I	1.29 × 109 (0-inf)	0.23
	Moderately differentiated	0.81 (0.42–1.55)	0.52		Grade II	1.31 (0.75–2.30)	0.35
	Well differentiated	–			Grade III	1.41 (0.93–2.14)	0.10

ADAMTS12	Poorly differentiated	1.30 (0.87–1.94)	0.21	ADAMTS12	Grade I	0.71 (0.04–11.79)	0.81
	Moderately differentiated	1.00 (0.52–1.92)	0.99		Grade II	1.50 (0.85–2.66)	0.16
	Well differentiated	0.95 (0.40–2.23)	0.90		Grade III	1.54 (1.02–2.34)	0.04

ADAMTS13	Poorly differentiated	0.87 (0.54–1.41)	0.58	ADAMTS13	Grade I	1.62 × 109 (0-inf)	0.32
	Moderately differentiated	1.06 (0.55–2.04)	0.87		Grade II	0.46 (0.25–0.85)	0.01
	Well differentiated	—			Grade III	0.88 (0.59–1.33)	0.56

ADAMTS14	Poorly differentiated	1.28 (0.78–2.08)	0.32	ADAMTS14	Grade I	1.29 × 109 (0-inf)	0.23
	Moderately differentiated	1.80 (0.93–3.47)	0.08		Grade II	0.81 (0.46–1.44)	0.48
	Well differentiated	—			Grade III	0.99 (0.66–1.49)	0.95

ADAMTS15	Poorly differentiated	0.97 (0.59–1.59)	0.91	ADAMTS15	Grade I	2.60 × 109 (0-inf)	0.09
	Moderately differentiated	1.34 (0.69–2.59)	0.38		Grade II	1.23 (0.70–2.17)	0.47
	Well differentiated	—			Grade III	1.59 (1.05–2.41)	0.03

ADAMTS16	Poorly differentiated	0.69 (0.42–1.14)	0.14	ADAMTS16	Grade I	0.71 (0.04–11.79)	0.81
	Moderately differentiated	1.43 (0.75–2.73)	0.28		Grade II	0.95 (0.54–1.68)	0.86
	Well differentiated	—			Grade III	1.56 (1.03–2.36)	0.03

ADAMTS17	Poorly differentiated	0.95 (0.59–1.53)	0.83	ADAMTS17	Grade I	1.41 (0.08–23.57)	0.81
	Moderately differentiated	1.14 (0.59–2.18)	0.70		Grade II	0.98 (0.55–1.74)	0.93
	Well differentiated	—			Grade III	0.77 (0.51–1.17)	0.22

ADAMTS18	Poorly differentiated	1.18 (0.73–1.91)	0.50	ADAMTS18	Grade I	1.29 × 109 (0-inf)	0.23
	Moderately differentiated	1.27 (0.66–2.43)	0.47		Grade II	1.27 (0.72–2.24)	0.41
	Well differentiated	—			Grade III	1.84 (1.20–2.83)	<0.01

ADAMTS19	Poorly differentiated	0.79 (0.49–1.28)	0.34	ADAMTS19	Grade I	1.86 × 109 (0-inf)	0.16
	Moderately differentiated	1.25 (0.65–2.38)	0.50		Grade II	1.12 (0.63–1.98)	0.71
	Well differentiated	—			Grade III	1.20 (0.79–1.81)	0.39

ADAMTS20	Poorly differentiated	1.10 (0.74–1.64)	0.63	ADAMTS20	Grade I	0 (0-inf)	0.09
	Moderately differentiated	0.95 (0.5–1.82)	0.88		Grade II	1.21 (0.68–2.15)	0.52
	Well differentiated	1.92 (0.81–4.57)	0.13		Grade III	1.20 (0.80–1.81)	0.38

**Table 3:** Correlation between genes expression of ADAMTS family and OS in gastric cancer patients of different genders.

ADAMTSs gene chip	Gender female (*n* = 244); male (*n* = 567)	HR (95% CI)	*P* value	ADAMTSs RNA-seq	Gender female (*n* = 133); male (*n* = 238)	HR (95% CI)	*P* value
ADAMTS1	Female	1.63 (1.05–2.50)	**0.03**	ADAMTS1	Female	1.37 (0.76–2.47)	0.29
	Male	1.63 (1.21–2.20)	**<0.01**		Male	1.39 (0.94–2.06)	0.10

ADAMTS2	Female	1.27 (0.83–1.95)	0.27	ADAMTS2	Female	1.59 (0.87–2.89)	0.13
	Male	1.68 (1.24–2.26)	**<0.01**		Male	1.16 (0.79–1.72)	0.45

ADAMTS3	Female	1.56 (1.09–2.22)	**0.01**	ADAMTS3	Female	1.27 (0.86–1.88)	0.23
	Male	1.24 (1.00–1.53)	0.05		Male	1.65 (0.90–3.02)	0.10

ADAMTS4 (Not find)	—			ADAMTS4	Female	0.86(0.48–1.54)	0.60
					Male	1.62(1.09–2.43)	**0.02**

ADAMTS5	Female	1.75 (1.13–2.70)	**0.01**	ADAMTS5	Female	1.65 (0.91–2.98)	0.10
	Male	1.47 (1.09–1.98)	**0.01**		Male	1.01 (0.68–1.49)	0.97

ADAMTS6	Female	1.57 (1.02–2.42)	**0.04**	ADAMTS6	Female	2.36 (1.28–4.35)	**<0.01**
	Male	1.76 (1.31–2.38)	**<0.01**		Male	1.47 (0.99–2.18)	0.05

ADAMTS7	Female	1.64 (1.07–2.52)	**0.02**	ADAMTS7	Female	1.35 (0.74–2.45)	0.32
	Male	1.88 (1.40–2.53)	**<0.01**		Male	1.48 (0.99–2.21)	0.05

ADAMTS8	Female	1.98 (1.28–3.08)	**<0.01**	ADAMTS8	Female	1.04 (0.58–1.86)	0.91
	Male	1.31 (0.97–1.76)	0.07		Male	1.16 (0.79–1.72)	0.45

ADAMTS9	Female	1.58 (1.03–2.43)	**0.03**	ADAMTS9	Female	1.72 (0.94–3.14)	0.08
	Male	1.27 (0.95–1.70)	0.11		Male	1.43 (0.97–2.13)	0.07

ADAMTS10	Female	1.08 (0.70–1.65)	0.73	ADAMTS10	Female	1.66 (0.91–3.01)	0.09
	Male	0.94 (0.70–1.26)	0.67		Male	1.26 (0.85–1.86)	0.25
ADAMTS12	Female	1.67 (1.17–2.37)	**<0.01**	ADAMTS12	Female	2.02 (1.10–3.70)	**0.02**
	Male	1.30 (1.05–1.61)	**0.02**		Male	1.63 (1.00–2.66)	**<0.05**

ADAMTS13	Female	0.94 (0.62–1.45)	0.79	ADAMTS13	Female	0.63 (0.35–1.15)	0.13
	Male	1.15 (0.86–1.54)	0.34		Male	0.87 (0.59–1.29)	0.49

ADAMTS14	Female	1.23 (0.80–1.89)	0.35	ADAMTS14	Female	1.09 (0.61–1.97)	0.76
	Male	1.80 (1.33–2.43)	**<0.01**		Male	1.00 (0.67–1.47)	0.98

ADAMTS15	Female	1.22 (0.79–1.87)	0.37	ADAMTS15	Female	0.86 (0.48–1.55)	0.61
	Male	1.23 (0.92–1.65)	0.16		Male	1.87 (1.25–2.79)	**<0.01**

ADAMTS16	Female	1.34 (0.87–2.05)	0.18	ADAMTS16	Female	1.83 (1.01–3.32)	**0.04**
	Male	1.08 (0.80–1.45)	0.61		Male	1.87 (1.25–2.79)	**<0.01**

ADAMTS17	Female	1.31 (0.85–2.01)	0.22	ADAMTS17	Female	0.78 (0.43–1.41)	0.41
	Male	1.12 (0.83–1.50)	0.45		Male	0.87 (0.59–1.29)	0.49

ADAMTS18	Female	1.32 (0.86–2.02)	0.21	ADAMTS18	Female	2.25 (1.23–4.10)	**<0.01**
	Male	1.43 (1.06–1.92)	**0.02**		Male	1.36 (0.92–2.02)	0.13

ADAMTS19	Female	1.22 (0.79–1.87)	0.36	ADAMTS19	Female	0.89 (0.49–1.60)	0.70
	Male	0.96 (0.72–1.30)	0.81		Male	1.26 (0.85–1.86)	0.25

ADAMTS20	Female	1.98 (1.38–2.83)	**<0.01**	ADAMTS20	Female	1.14 (0.64–2.06)	0.65
	Male	1.67 (1.35–2.07)	**<0.01**		Male	1.12 (0.75–1.65)	0.58

## Data Availability

The data used to support the findings of this study are available from the corresponding author upon request.
